# A simple method for generating high-resolution maps of genome-wide protein binding

**DOI:** 10.7554/eLife.09225

**Published:** 2015-06-16

**Authors:** Peter J Skene, Steven Henikoff

**Affiliations:** 1 Fred Hutchinson Cancer Research Center, Seattle, United States; 2 Howard Hughes Medical Institute, Fred Hutchinson Cancer Research Center, Seattle, United States; University of Colorado at Boulder, United States

**Keywords:** transcription factors, RNA polymerase II, CTCF, *D. melanogaster*, human

## Abstract

Chromatin immunoprecipitation (ChIP) and its derivatives are the main techniques used to determine transcription factor binding sites. However, conventional ChIP with sequencing (ChIP-seq) has problems with poor resolution, and newer techniques require significant experimental alterations and complex bioinformatics. Previously, we have used a new crosslinking ChIP-seq protocol (X-ChIP-seq) to perform high-resolution mapping of RNA Polymerase II (Skene et al., 2014). Here, we build upon this work and compare X-ChIP-seq to existing methodologies. By using micrococcal nuclease, which has both endo- and exo-nuclease activity, to fragment the chromatin and thereby generate precise protein–DNA footprints, high-resolution X-ChIP-seq achieves single base-pair resolution of transcription factor binding. A significant advantage of this protocol is the minimal alteration to the conventional ChIP-seq workflow and simple bioinformatic processing.

**DOI:** http://dx.doi.org/10.7554/eLife.09225.001

## Main text

**Figure 1.**
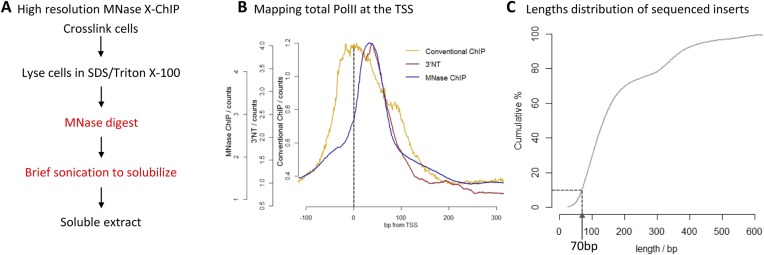
High-resolution X-ChIP-seq of PolII at transcriptional start sites (TSS). (**A**) Experimental workflow using MNase to fragment the chromatin. (**B**) Average PolII profile across TSS in Drosophila. S2 cells as measured by conventional ChIP (Core et al., 2012), high-resolution X-ChIP-seq (fragment lengths 20–70 bp) and 3′NT that maps the position of the polymerase active site via the last ribonucleotide incorporated into the nascent chain (Weber et al., 2014). With 3′NT, the RNA has to be transcribed at least 25 nt in length to be mapped. (**C**) Length distribution of the mapped paired-end reads from a total PolII high-resolution X-ChIP-seq experiment (Skene et al., 2014). **DOI:** http://dx.doi.org/10.7554/eLife.09225.002

The diverse gene expression programs that allow for differentiation and response to environmental stimuli result from the regulated binding of transcription factors to DNA. The prevalent technique used in chromatin biology for mapping protein–DNA interactions is chromatin immunoprecipitation (ChIP), but little has changed since it was first described 27 years ago (Solomon et al., 1988). Despite recent advances in read-out technologies for ChIP, such as high-throughput sequencing (ChIP-seq), the basic chromatin preparation protocol remains the same and has a number of limitations. For example, sonication is typically used to fragment the chromatin. This however, has been shown to be non-random, with heterochromatic regions showing increased resistance to fragmentation leading to bias in the experiment (Teytelman et al., 2009). In addition, sonication typically produces chromatin fragments between 200 and 500 bp, whereas the footprint of a typical chromatin-associated protein is ∼10-fold smaller, indicating a lack of resolution currently obtained by ChIP-seq. Even extensive sonication only yields fragments with an average length of 200 bp, suggesting that sonication is of limited use in generating high-resolution maps of genome-wide protein binding (Fan et al., 2008). In a previous study, we were interested in how RNA Polymerase II transcribes through nucleosomes at the promoter (Skene et al., 2014). Answering this question required the precise mapping of PolII with respect to the position of nucleosomes, but conventional ChIP-seq that uses sonication to fragment the chromatin, yields fragments approximately twice the size of a nucleosome. Additionally, it has been shown that PolII can crosslink to nearby nucleosomes and therefore mapping the immunoprecipitated DNA fragments from these composite PolII:nucleosome:DNA complexes fails to precisely map the position of PolII on the DNA (Koerber et al., 2009; Skene et al., 2014). By using micrococcal nuclease (MNase) to digest unprotected DNA, we were able to achieve high resolution in a ChIP experiment, mapping the precise location of PolII and chromatin remodelers on the DNA (Figure 1A; a detailed protocol is provided as a Supplementary file 1) (Skene et al., 2014). Optimization of this simple protocol for the high-resolution mapping of protein–DNA interactions has the potential to revolutionize our understanding of genome-wide protein binding. High resolution is especially a requirement at closely spaced transcription factor binding sites, such as locus control regions and super-enhancers, which have been shown to be vital to cell fate decisions and human diseases (Hnisz et al., 2013; Pott and Lieb, 2014).

**Figure 1—figure supplement 1.**
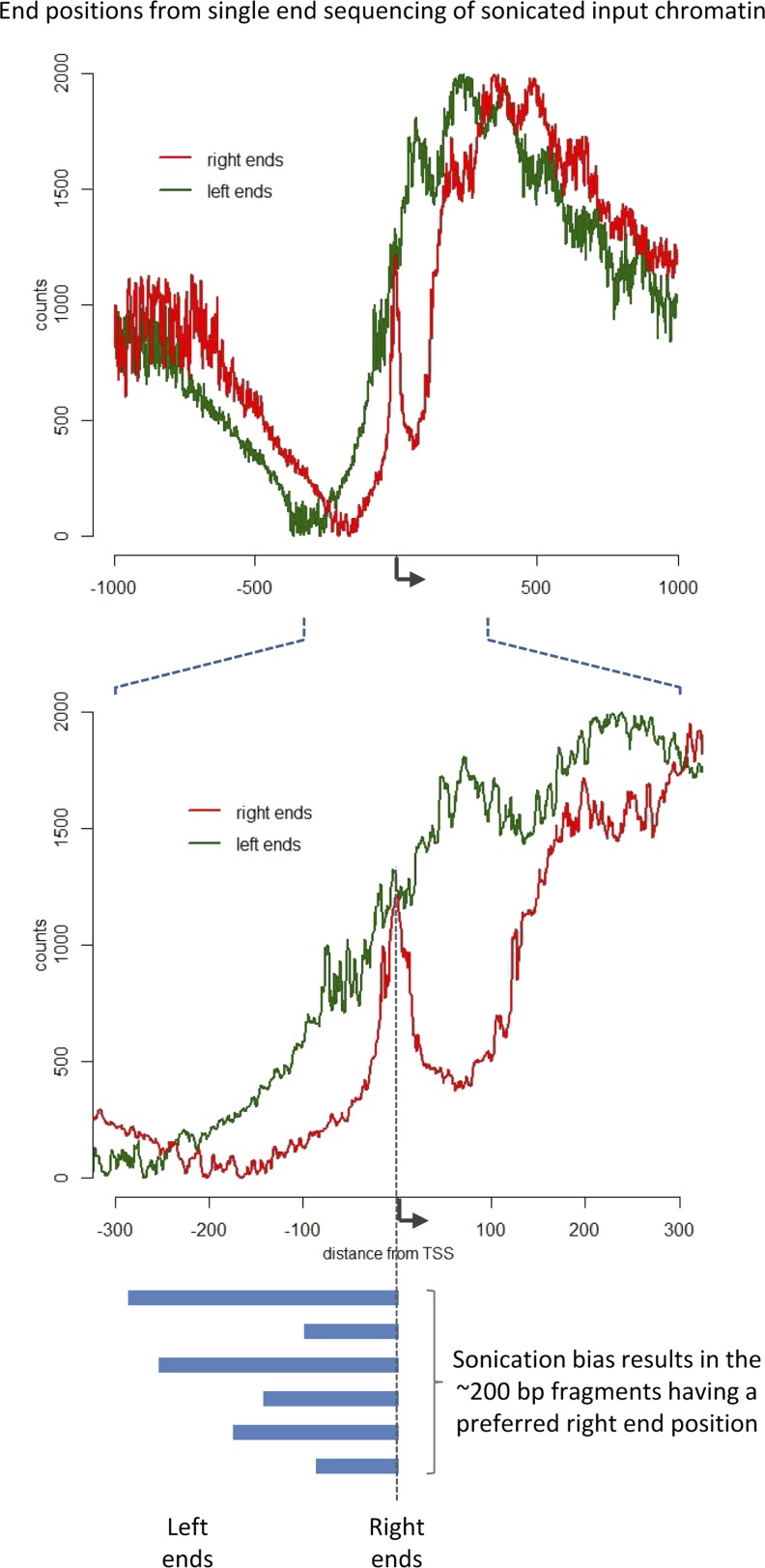
Sonication bias at promoter regions. The left ends (5′ position on forward strand reads) and right ends (3′ position on reverse strand reads) of fragments from single-end sequencing data of sonicated input chromatin was aligned to the TSS (reads corresponding to genes on the reverse strand were flipped). The non-uniform distribution indicates a bias with the distinct peak at the TSS arising from non-random chromatin fragmentation, with a high probability of having a right end just downstream of the TSS. The input data set from modENCODE ID#3953 (GSE47229) was used. Due to the sonication bias, it is clear that the sonicated fragments do not represent the minimally protected DNA footprint, and as such the length of the recovered DNA fragments provides no information. **DOI:** http://dx.doi.org/10.7554/eLife.09225.003

To evaluate high-resolution crosslinking ChIP-seq (X-ChIP-seq), we compared it to existing methodologies. Initially, we focused on mapping PolII at the transcriptional start site (TSS) in *Drosophila* S2 cells, where there are existing data sets at both low and high resolution. We performed high-resolution X-ChIP-seq using the same antibody against total PolII (Rpb3 subunit) as previously used by a conventional sonication ChIP experiment (Figure 1B) (Core et al., 2012). Using this cell line also allowed a comparison with the single base-pair resolution technique that maps the last ribonucleotide incorporated into the nascent RNA chain (3′NT), thereby mapping the exact position of the PolII active site (Weber et al., 2014). By using paired-end sequencing, we can selectively study specific lengths of immunoprecipitated fragments. Analyzing sequenced fragments with lengths 20–70 bp, which more closely represent the footprint of PolII (Samkurashvili and Luse, 1996), avoids the complication of mapping fragments consisting of PolII crosslinked to adjacent nucleosomes. Using this technique, we find that the maximal peak of PolII signal coincides with the position of the polymerase's active site at ∼+35 bp, as measured by 3′NT. This is consistent with evidence suggesting that the vast majority of *Drosophila* genes have a productively engaged PolII enzyme stalled just downstream of the promoter rather than PolII stably bound at the pre-initiation complex (Core et al., 2012). In contrast, PolII distribution as measured by conventional ChIP with the chromatin fragmented by sonication, shows a distinct distribution at the promoter with a broader peak centered at the TSS with maximal density at −5 bp. This discrepancy likely comes from biases in the probability of sonication breaking the DNA at the nucleosome-depleted region of the promoter, as accessible regions such as DNase I sites and promoters of active genes have been shown to be sonicated at higher probability than inactive genomic regions (Teytelman et al., 2009). Analysis of a published sonicated input chromatin sample indicates a strong sonication bias at the promoter region (Figure 1—figure supplement 1). In contrast, by predominantly fragmenting the chromatin with MNase, it is possible to generate footprints corresponding to nucleosomes and other DNA-bound factors (Henikoff et al., 2011; Skene et al., 2014). Overall, this shows that using a high-resolution ChIP technique to map the protected footprint of PolII achieves comparable resolution to the single base-pair resolution achieved by mapping the position of the active site of PolII via nascent chain mapping. In comparison to conventional ChIP-seq, using high-resolution X-ChIP-seq achieves both higher resolution, as indicated by the width of the ChIP peak and higher accuracy by avoiding sonication bias, as shown by high similarity to 3′NT. Moreover, the depth of sequencing indicates the cost-effectiveness of this high-resolution ChIP approach, with the 3′NT profile based on 150 million reads (Weber et al., 2014), whereas our method required only 7 million paired-end reads with a fragment length of 20–70 bp. For comparison, the PolII profile generated by conventional ChIP was based on 13 million mapped reads (Core et al., 2012).

**Figure 2.**
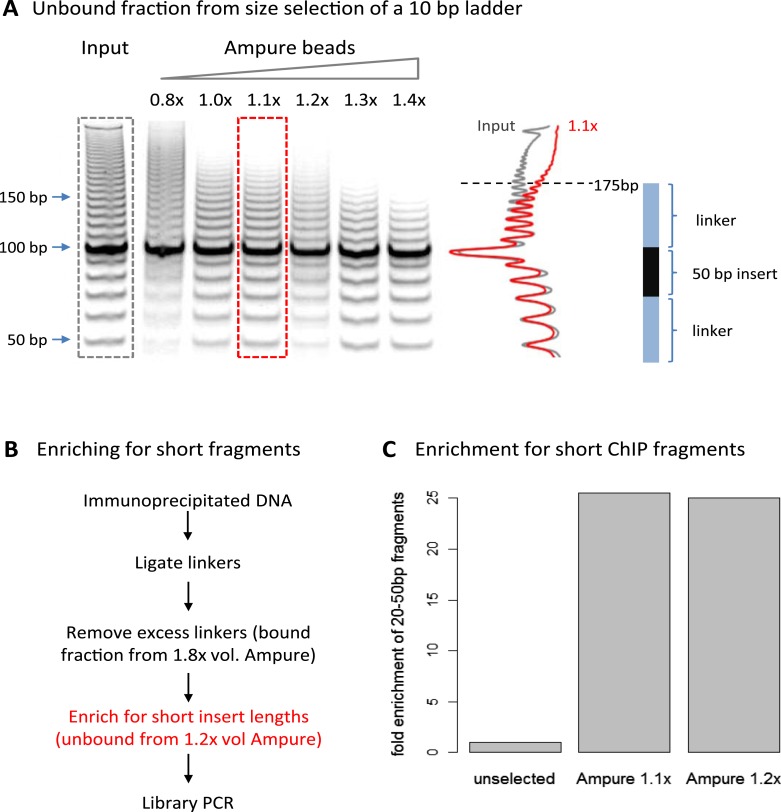
Size selection to enrich for short immunoprecipitated fragments. (**A**) Volumetric ratio of AMpure beads to DNA was optimized to selectively retain fragments below 200 bp in the unbound fraction using a 10-bp ladder as a test case. The cartoon indicates the size of ligated product containing a 50-bp insert. (**B**) Library preparation workflow to enrich for short insert sizes between the ligated linkers. (**C**) Fold enrichment of sequenced ChIP fragments less than 50 bp after the AMpure size selection as depicted in panel **B**. This method of enriching for short fragments is specifically applicable to high-resolution X-ChIP-seq, where MNase has been used to generate minimally protected DNA footprints. In contrast, in conventional ChIP-seq where sonication is used, the enrichment of short size classes would be inappropriate as typically fragments are between 200 and 500 bp in length and even extensive sonication can only further fragment chromatin to an average size of 200 bp (Fan et al., 2008). **DOI:** http://dx.doi.org/10.7554/eLife.09225.004

A limitation of high-resolution X-ChIP-seq is that a minority of the immunoprecipitated fragments represent the footprint of PolII on DNA, likely as a consequence of formaldehyde readily forming protein–protein crosslinks generating complexes such as PolII crosslinked to nucleosomes (Koerber et al., 2009; Skene et al., 2014). In our previous study, mapping murine PolII, only 10% of the fragments were 20–70 bp in length and less than 3% were under 50 bp (Figure 1C) (Skene et al., 2014). Therefore, to improve the cost-effectiveness of this technique and make it more applicable to transcription factors, which typically have a <50-bp footprint, we have further optimized the method to enrich for short fragments prior to sequencing. Previously, Agencourt AMpure beads have been used to select for short fragments prior to linker ligation (Orsi et al., 2015). In agreement, initial attempts indicated that Agencourt AMpure beads could enrich for DNA fragments below 100 bp from a complex mixture, but were unable to selectively purify fragments of ∼50 bp. However, by adjusting the volumetric ratio of beads to DNA, we could reproducibly control the selection within the 100–200 bp range with a ratio of 1.1×, leaving fragments of ∼170 bp in the unbound fraction (Figure 2A). Given that the ligation of the Illumina adapters to the immunoprecipitated DNA adds ∼125 bp, by using this ratio of AMpure beads, we could selectively enrich for ligated products containing short inserts (Figure 2B). Using this approach on input DNA from a MNase ChIP experiment, where the vast majority of the DNA fragments are from mono-nucleosomes, we find a 25-fold enrichment of fragments below 50 bp (Figure 2C). Therefore, combining this modification to the existing library preparation protocol with the MNase X-ChIP approach yields cost-effective high-resolution data.

**Figure 3.**
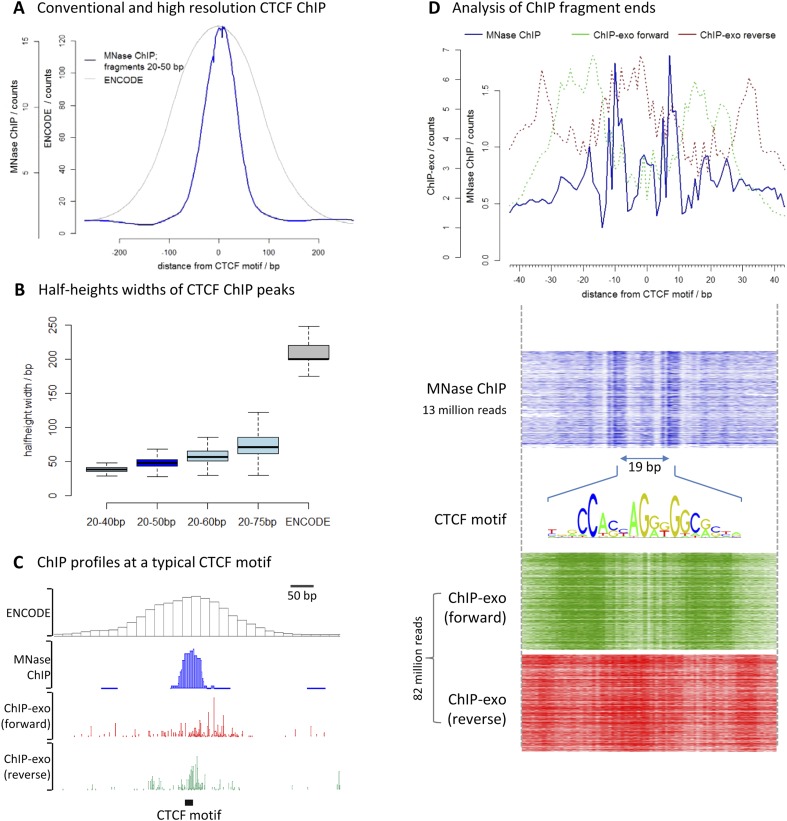
High-resolution X-ChIP-seq provides base-pair resolution of the minimal CTCF sequence motif. (**A**) Average CTCF profile at DNase I sites that contain the motif in K562 cells, as measured by conventional ChIP in the ENCODE project and high-resolution X-ChIP-seq (20–50 bp fragments). Sites were determined by identifying the DNase I sites common to K562 and HeLa cells, as defined by the ENCODE project, that contained the 19 bp CTCF consensus binding motif (MA0139.1) by using FIMO analysis with a false discovery rate of 0.01 (Grant et al., 2011). This identified 9403 such 19 bp CTCF motifs within DNase I sites that were at least 500 bp apart. (**B**) Box plots indicating half-height widths of ChIP peaks at each individual CTCF motif for different size classes of immunoprecipitated fragments in high-resolution X-ChIP-seq and conventional ChIP. (**C**) ChIP profiles at a typical CTCF motif. For ChIP-exo, the 5′ ends of forward and reverse strands are plotted. (**D**) The upper graph displays the average profile mapping the position of both of the ends of paired-end reads for the 20–50 bp immunoprecipitated CTCF fragments in high-resolution X-ChIP-seq centered over the CTCF motif. For comparison, the ends for the forward and reverse strands are shown for ChIP-exo. The heatmaps below show the signal ±40 bp for each CTCF motif (defined as CTCF motifs with DNase I sites in both HeLa and K652 cells; n = 9403). The 19 bp between the identified peaks is highlighted and the 19 bp CTCF motif indicated. **DOI:** http://dx.doi.org/10.7554/eLife.09225.005

To assess the resolution of this method, we chose the well-characterized transcription factor CCCTC-binding factor (CTCF). We performed high-resolution X-ChIP-seq in K562 cells and analyzed 20–50 bp fragments, and compared this to conventional ChIP as used in the ENCODE project (Figure 3A). To avoid the complexities of peak-calling algorithms that might be biased for differing data types, we used an unbiased approach of centering the data on CTCF motifs that were found within DNase I sites and therefore most likely bound by CTCF. High-resolution X-ChIP-seq of CTCF yielded a more focused distribution of reads centered over the CTCF motif. To quantify this, we measured the width of the ChIP peak at its half-height for each individual CTCF site (Figure 3B). A conventional ChIP approach using sonication gave a half-height width of 200 bp. In contrast, analysis of 20–50 bp fragments from MNase ChIP gave much higher resolution, with a half-height width of only 50 bp, suggesting that genome-wide MNase is chewing back to a minimal footprint of CTCF bound to the DNA. By analyzing different ranges of fragment lengths, it was possible to see that shorter fragments gave the highest resolution and smallest range in peak widths (Figure 3B and Figure 3—figure supplement 1).

**Figure 3—figure supplement 1.**
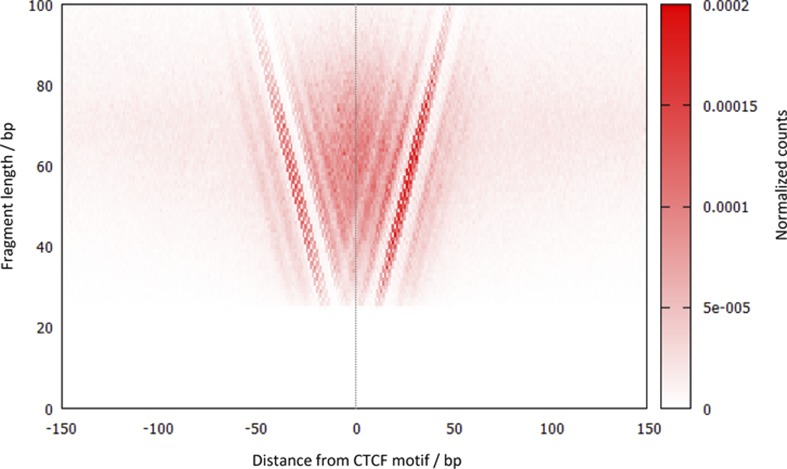
V plot of DNA fragments recovered by CTCF high-resolution X-ChIP-seq. The V-plot is a midpoint-vs-length map centered on the aligned CTCF motifs (n = 9403), where a dot is placed on a 2D map with the x-axis representing the midpoint position of each immunoprecipitated fragment and the y-axis representing the length of that DNA fragment (Henikoff et al., 2011). Normalized counts represent the number of fragments at each pixel position relative to the total number of pixels. Note that the shorter DNA fragments are more tightly grouped and closely centered over the CTCF motif. **DOI:** http://dx.doi.org/10.7554/eLife.09225.006

**Figure 3—figure supplement 2.**
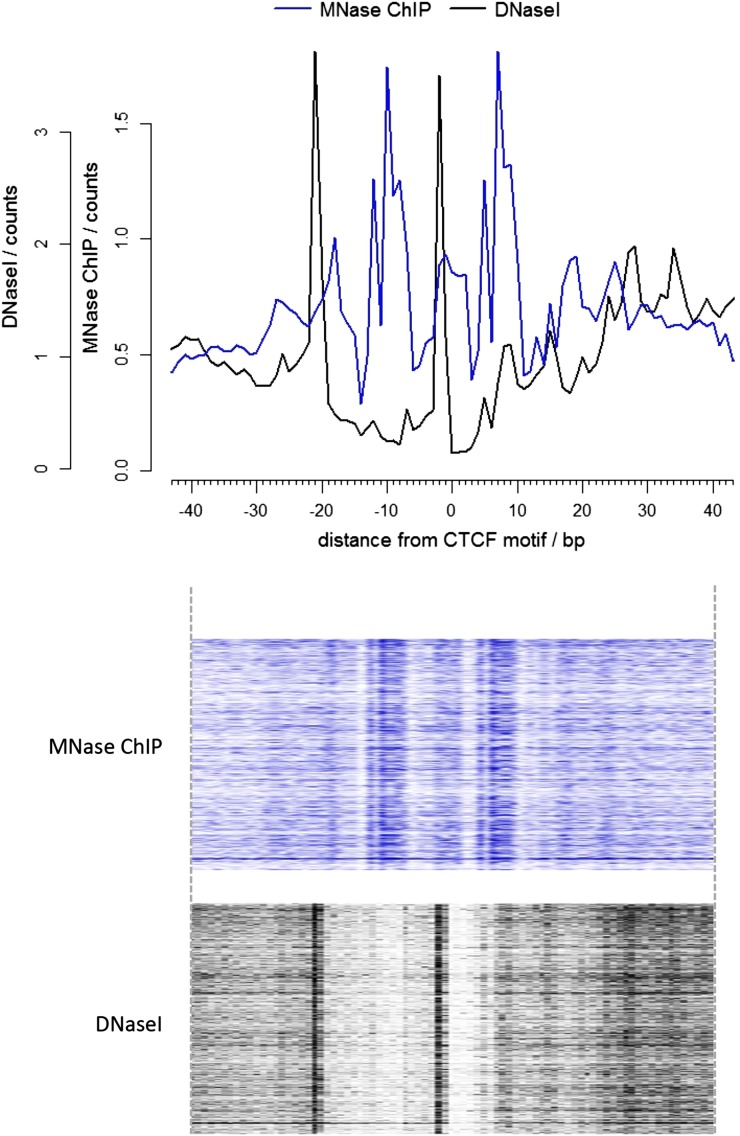
Comparison of the ends of DNA fragments from DNase I and high-resolution X-ChIP-seq centered over the CTCF motif. The upper graph shows the average profile, and the heatmaps below show the signal ±40 bp for each CTCF motif. The leftward shift of the DNase I footprint likely reflects differences in the steric interactions between CTCF and DNase I and that of MNase. **DOI:** http://dx.doi.org/10.7554/eLife.09225.007

We also compared our CTCF high-resolution X-ChIP-seq results to CTCF profiles obtained using ChIP-exo, which is based upon the sonication of crosslinked chromatin, followed by exonuclease digestion of the immunoprecipitated complexes (Rhee and Pugh, 2011). In ChIP-exo, sequential ligation reactions allow the demarcation of 5′ and 3′ ends and bioinformatic analysis is used to identify ‘peak pairs’ that flank the transcription factor binding site. Figure 3C shows profiles based on ENCODE X-ChIP-seq data as processed by the ENCODE uniform processing pipelines and downloaded as ‘raw signal’, our high-resolution X-ChIP-seq stacked read data, and raw ChIP-exo data around a representative CTCF motif. Due to the low amounts of noise, high-resolution X-ChIP-seq is amenable to a very simple thresholding algorithm to identify peaks (Kasinathan et al., 2014), requiring only 13 million paired-end reads to obtain a crisp peak feature. This is in contrast to ChIP-exo, where more complex analysis is required, including dedicated software (Rhee and Pugh, 2011; Starick et al., 2015). Based on 82 million reads, the ChIP-exo raw data show significant signal at a distance from the CTCF motif. This might be a consequence of immunoprecipitating sonicated 200–300 bp chromatin fragments containing more than one protein, which would block the subsequent exonuclease cleavage. However, by using MNase to fragment the chromatin, which has both endo- and exo-nuclease activity, high-resolution X-ChIP-seq should be able to discriminate between nearby proteins. An additional limitation of ChIP-exo is that the input chromatin is not subjected to the same exonuclease treatment and therefore subsequent analyses cannot be normalized to input. With high-resolution X-ChIP-seq, however, all the steps in chromatin fragmentation are prepared prior to immunoprecipitation, thereby allowing input normalization. Moreover, high-resolution X-ChIP-seq requires only minimal alteration to the existing conventional ChIP workflow and library preparation, whereas other techniques require more extensive changes (Starick et al., 2015). To more directly compare to ChIP-exo, we plotted the end positions of each of our 20–50 bp paired-end reads (Figure 3D). We find two predominant sharp peaks on either side of the 19-bp CTCF motif that are separated by 19 bp, indicating that on average for each of our immunoprecipitated fragments, MNase has chewed back to one side of the minimal sequence motif. In contrast, the signal for ChIP-exo is relatively diffuse when centered around the CTCF motif, with an average distance of 52 bp between peak pairs for the peak-called sites (Rhee and Pugh, 2011). DNase I footprinting is often used to generate maps of global transcription factor binding at nucleotide resolution, with the drawback that the technique is not targeted to a specific transcription factor (Hesselberth et al., 2009; Neph et al., 2012). By comparing the ends of the DNA fragments released by DNase I footprinting and that of high-resolution X-ChIP-seq, we find that both techniques identify protected fragments with ends separated by 19 bp at the 19 bp consensus CTCF motifs (Figure 3—figure supplement 2). This therefore suggests that high-resolution X-ChIP-seq can achieve single nucleotide resolution, and by using immunoprecipitation, has the advantage that it can be used to interrogate individual transcription factors.

**Figure 4.**
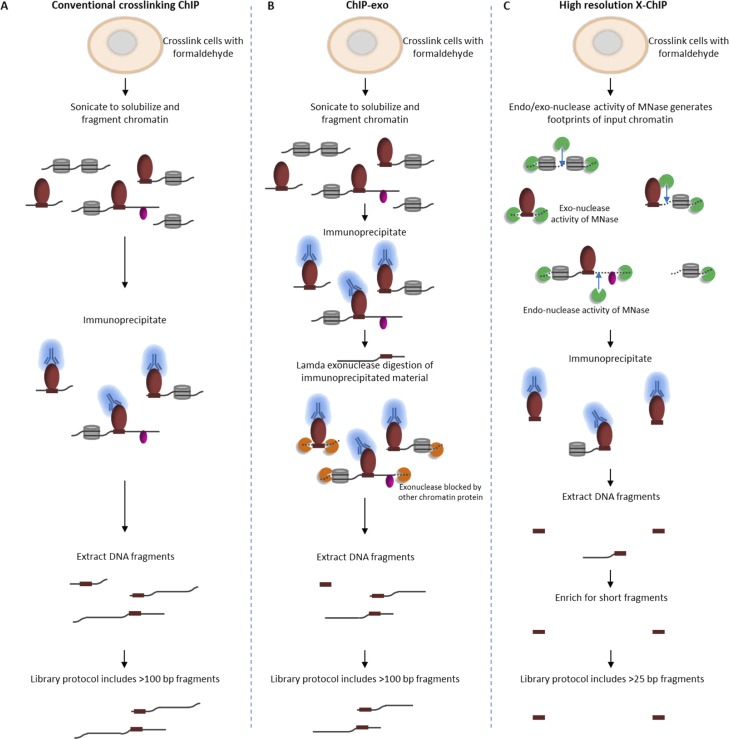
Comparison of different ChIP methodologies and how the resolution obtained depends on the fragmentation strategy used. The fragmentation strategy is shown for (**A**) conventional ChIP-seq, (**B**) ChIP-exo and (**C**) high-resolution X-ChIP-seq. In high-resolution X-ChIP-seq, MNase generates minimally protected DNA fragments that are represented by the lengths of the extracted DNA fragments, which can be obtained by paired-end sequencing. By using an AMpure size selection, it is possible to enrich for these short fragments and increase the cost-effectiveness of the technique. In contrast, conventional ChIP and ChIP-exo are designed for single-end sequencing. Furthermore, the protocols used to generate sequencing libraries for conventional ChIP-seq and ChIP-exo select against fragments below 100 bp. **DOI:** http://dx.doi.org/10.7554/eLife.09225.008

By harnessing the endo- and exo-nuclease activity of MNase to fragment chromatin, high-resolution X-ChIP-seq has key advantages over conventional ChIP-seq and ChIP-exo in terms of the resolution obtained (Figure 4). Overall, the combination of the improvements to enrich for short immunoprecipitated fragments and the unparalleled ChIP resolution for PolII and transcription factor binding indicate that high-resolution X-ChIP-seq is a cost-effective and simple approach that easily fits within existing ChIP-seq pipelines for determining precise genome-wide maps of protein–DNA binding.

## Materials and methods

### Cell lines

*Drosophila* S2 cells and K562 cells were cultured under standard conditions.

### ChIP

High-resolution X-ChIP-seq was performed as described in the Supplementary file 1. Libraries were prepared from the isolated DNA and following cluster generation, 25 rounds of paired-end sequencing was performed by the FHCRC Genomics Shared Resource on the Illumina HiSeq 2500 platform (Henikoff et al., 2011). Details of the library protocol have previously been published (Orsi et al., 2015). After processing and base-calling by Illumina software, paired-end sequencing data were aligned to the hg19 or dmel_r5_51 genome build using Bowtie or Novoalign, respectively. Counts per base pair were normalized as previously described with the fraction of mapped reads spanning each base-pair position multiplied by the genome size (Kasinathan et al., 2014). To analyze reads by length, we divided paired-end fragments into distinct size classes, as indicated in the figure legends. V-plot construction has been previously described (Henikoff et al., 2011). Half-height width for each individual site was calculated as follows: the half height was calculated by dividing the maximum ChIP signal within ±1000 bp of each 19 bp CTCF motif by the background signal, which was defined as the median ChIP signal between −1000 to −900 and +900 to +1000 bp relative to the motif. The half-height width for each motif was calculated by counting the number of contiguous base pairs that had ChIP signal greater than or equal to the half-height.

## Additional files

Supplementary file 1. Protocol for high-resolution X-ChIP-seq. Detailed protocol for performing high-resolution X-ChIP-seq in cell lines. **DOI:** http://dx.doi.org/10.7554/eLife.09225.009

### Major datasets

The following datasets were generated:

Skene PJ, Henikoff S, 2015, A simple method for generating high-resolution maps of genome wide protein binding, http://www.ncbi.nlm.nih.gov/geo/query/acc.cgi?acc=GSE67454, Publicly available at the NCBI Gene Expression Omnibus (Accession no: GSE67454).

Skene PJ, Hernandez AE, Groudine M, Henikoff S, 2014, The nucleosomal barrier to promoter escape by RNA Polymerase II is overcome by the chromatin remodeler Chd1, http://www.ncbi.nlm.nih.gov/geo/query/acc.cgi?acc=GSE52349, Publicly available at the NCBI Gene Expression Omnibus (Accession no: GSE52349).
