# Supplementary material for: A simple method for generating high-resolution maps of genome-wide protein binding
Source: eLife. 2015 Jun 16;4:e09225. doi: 10.7554/eLife.09225 (PMC4480131; doi:10.7554/eLife.09225)
Supplement: Supplementary file 1. — Protocol for high-resolution X-ChIP-seq. Detailed protocol for performing high-resolution X-ChIP-seq in cell lines. DOI: http://dx.doi.org/10.7554/eLife.09225.009 [file elife09225s001.pdf]

X-link ChIP (MNase followed by sonication)

Protocol based upon the MNase digestion of crosslinked/lyzed cells to produce protected fragments. A brief pulse of sonication is then used to ensure near-complete extraction of chromatin associated proteins. The extent of MNase digestion is such that typically 90% mononucleosomes are observed by EtBr-agarose gel. In addition, a large population of heterogeneously sized sub-nucleosomal fragments are observed.

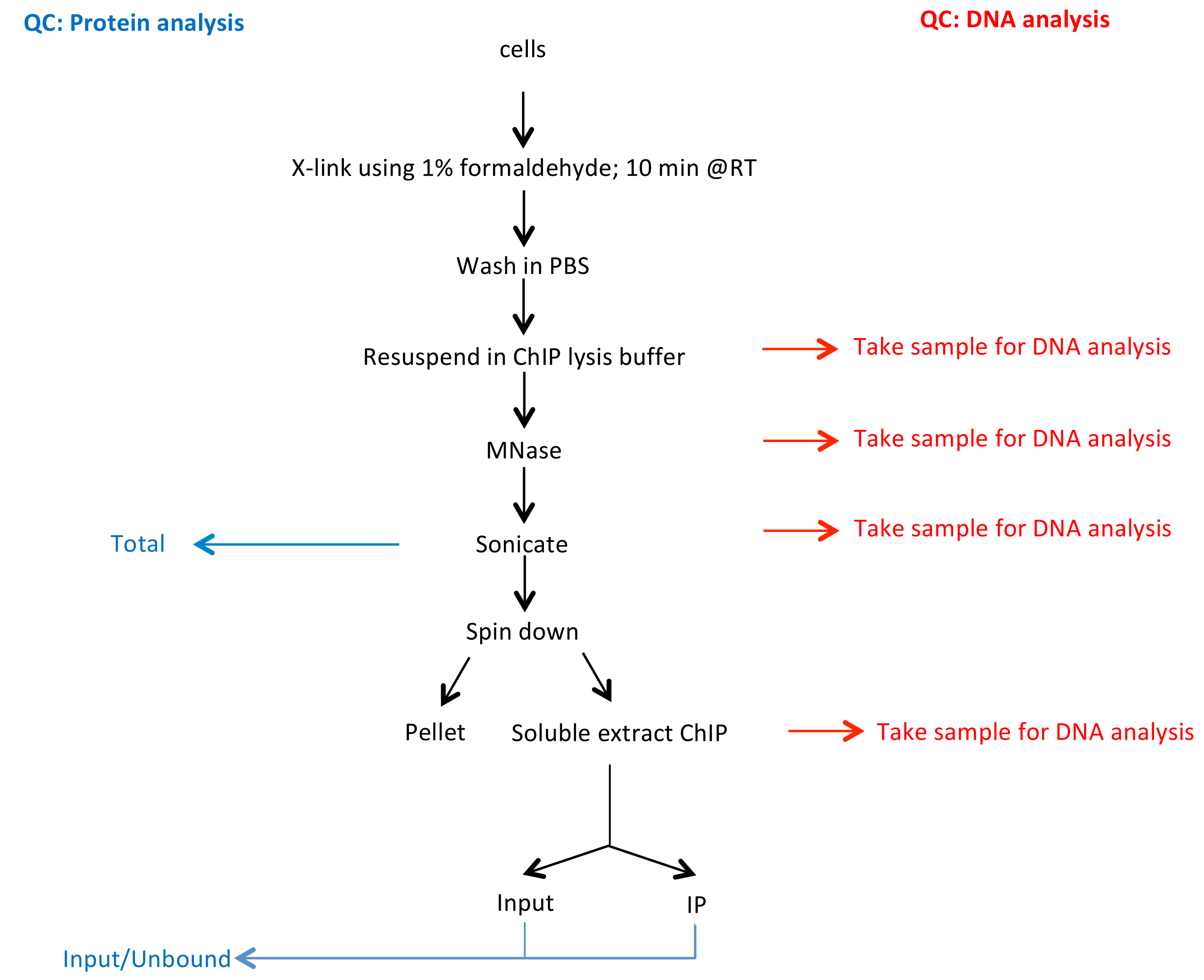

Typically perform fragmentation on 5-10 x 10^6 cells in the 1.5ml reaction volume, which can then be divided to the multiple IPs. MNase amount needs to be determined empirically for each cell type.

Abbreviations:

PIs protease inhibitors; typically prepared as 50x solution from Roche tablet (Cat #06538282001). Added to a final concentration of 1x

1) Trypsinise/quench cells with media; count

2) Spin down cells (3 min, 600g) in swing bucket rotor in 15 ml falcons

3) Wash in 2ml ice-cold PBS + PIs; resuspend by pipetting (never vortex); spin as above

4) Resuspend cells in 1ml PBS (at room temp)
   - Add 13 ml 1.077% formaldehyde in room temp PBS (prepare master mix for all samples, e.g. 2.02ml 16% formaldehyde + 27.98 ml room temp PBS)
   - *Using methanol free 16% formaldehyde stock from Polysciences #18814*
   - Incubate 10 min at room temp on wheel
   - Quench by adding glycine to 125 mM (0.7 ml of 2.5 M); ~2 min at room temp
   - Spin down as above
   -

5) Wash in 2ml ice-cold PBS + PIs; resuspend by pipetting (never vortex);
   - transfer to Eppendorf
   - spin as above (*I prefer swing bucket centrifugation as gives a better pellet)*

6) Resuspend in 150 µl ice cold lysis buffer + PIs; 10 min on ice *(This is for a typical fragmentation with 5-10 x 10^6 cells. For higher cell counts, scale up the lysis, dilution and Stop buffers and perform the MNase digestion in a single larger reaction volume. After stopping the MNase reaction (step 9), separate the into multiple 1.5 ml reactions for the sonication)*

7) To each tube, add 1350 µl supplemented *ice cold* ChIP dilution buffer (add PIs + 3 mM $CaCl_2$).
   - Take 40 µl for DNA analysis

8) Place tubes at 37C for 2 min to pre-warm. Add 5µl MNase (lab stock); @ 37 C for 15 min
   (the amount of MNase will need to be determined empirically for each cell type in order to generate ~75% mononucleosomes; see PMID: 24737864 Figure 1—figure supplement 1.)

9) Stop by adding 30 µl EDTA (stock 500 mM; final conc 10mM) and 60 µl EGTA (stock 500 mM; final conc 20mM). Place on ice and mix thoroughly by inverting.
   (Prepare a master mix of stop solution to allow quicker addition)
   - Transfer to 15 ml falcon
   - Take 40 µl for DNA analysis

10) Sonicate using Branson digital sonifier: 40s on time; 30%; 2.5s on; 5s off
   - Use EtOH/ice bath during sonication
   - This represents "Total": Take 20 µl for protein analysis + 10 µl 2 x SDS LB
     Take 40 µl for DNA analysis

11) Spin down (2min @ 16000 x g; 4C) to produce soluble extract
   - Take S/N as Input: Take 40 µl for DNA analysis

Optional step:

1) *Pre-block beads with BSA and tRNA*
2) *Pre-clear soluble chromatin after step 11 with the blocked beads to reduce background*

DNA extraction for QC samples taken *during* protocol:

Prepare extraction buffer

1 rxn	
3 µl	Tris-HCl pH8.0 (1M; final 10 mM)
6 µl	EDTA (500 mM; final 10 mM)
12 µl	EGTA (500 mM; final 20 mM)
18 µl	NaCl (5 M; stock 300 mM)
5 µl	Proteinase K
30 µl	SDS (10%; final 1%)
186 µl	H_20
260 µl	

Add the 40 µl DNA analysis samples to this and de-crosslink overnight at 65C

Extract with phenol:chloroform the next day and EtOH ppt

Resuspend in H_2O and RNase treat before running on gel

12) Continue with ChIP by combining and mixing any soluble chromatin extracts as required (e.g. did multiple 10 million cell fragmentations as have lots of separate IPs)

- Take sample for Input (~200 µl)
- Take sample for IP (~1200 µl)
- Place on wheel overnight at 4C

Space for notes on volumes of input/IPs and details of antibodies

After overnight incubation spin down (2min @ 16000 x g; 4C) to remove any proteins that precipitated overnight. This should be done to both IPs and Inputs. This step can't be used if using conjugated anibody/beads.

13) Next day take 20 µl of Input + 10 µl 2 x SDS LB
    - Store at -20 C

14) Add beads to IPs, on wheel for 1h at 4C; keep inputs on ice until extraction

15) Use magnet to capture beads or spin down depending on beads used.
    - Take 20 µl of unbound + 10 µl 2 x SDS LB; store at -20 C

16) wash with 1 ml of ice cold buffers with a ~2 min incubation on wheel at room temp

    1 x TSE1
    4 x TSE2
    1 x Buffer 3
    3 x TE

17) Extract directly from the beads by incubating with Proteinase K and decrosslinking
    - After the last wash, resuspend beads in 300µl extraction buffer, add extraction buffer to input to bring volume to total 300µl
    - Incubate at 65C overnight

1 rxn	*Extraction buffer*
6 µl	1 M Tris pH 8.0 (final 20 mM)
6 µl	0.5 M EDTA (final 10 mM)
7.5 µl	0.2 M EGTA (final 5 mM)
30 µl	10% SDS (final 1%)
18 µl	5M NaCl (final 300 mM)
3 µl	Proteinase K
229.5	H_2O
300 µl	

18) Phenol/CCl3/IAA extract once with 300 µl

19) - EtOH precipitate: add 850 µl EtOH + 2 ul glycogen
    - place at -80 C for at least 30 min
    - fast spin for ~30 min at 4 C
    - wash pellet with 800 µl 70 % EtOH
    - allow pellets to air dry for ~10 min; resuspend in 0.1 x TE (pH8.0)

Buffers:

Lysis Buffer:
1% SDS
10 mM EDTA
50 mM Tris-HCl (pH 8.1)

IP dilution Buffer:
1% Triton X-100
2 mM EDTA
150 mM NaCl
20 mM Tris-HCl (pH 8.1)

TSE I:
0.1% SDS
1% Triton X-100
2 mM EDTA
20 mM Tris-HCl (pH 8.1)
150 mM NaCl

TSE II:
0.1% SDS
1% Triton X-100
2 mM EDTA
20 mM Tris-HCl (pH 8.1)
500 mM NaCl

Buffer III:
0.25 M LiCl
1% NP-40
1% deoxycholate
1 mM EDTA
10 mM Tris-HCl (pH 8.1)
