# Supplementary material for: A simple method for generating high-resolution maps of genome-wide protein binding
Source: eLife. 2015 Jun 16;4:e09225. doi: 10.7554/eLife.09225 (PMC4480131; doi:10.7554/eLife.09225)
Supplement: Related texts

# Article commentary: Decision letter

**DOI:** 10.7554/eLife.09225.010

**Affiliations:** University of Colorado at Boulder, United States

eLife posts the editorial decision letter and author response on a selection of the published articles (subject to the approval of the authors). An edited version of the letter sent to the authors after peer review is shown, indicating the substantive concerns or comments; minor concerns are not usually shown. Reviewers have the opportunity to discuss the decision before the letter is sent (see review process). Similarly, the author response typically shows only responses to the major concerns raised by the reviewers.

Thank you for submitting your work entitled “A simple method for generating high‐resolution maps of genome wide protein binding” for peer review at *eLife*. Your submission has been reviewed by James Manley (Senior editor) and a Reviewing editor. We feel the work as it stands is not yet fully developed to a level that we could consider for publication as a Research Advance in *eLife*. However, if you are able to address these concerns with additional data and significant revisions of the text to include more detail, we would be prepared to consider a resubmission on this topic that would be evaluated by the same editors.

The authors present modifications to the widely used ChIP-seq technique as a new method entitled X-ChIP-seq. These modifications simply involve the addition of a micrococcal nuclease digestion step to reduce background DNA fragments and a size-selection step to enrich for short fragments prior to sequencing. It is claimed that these modifications both greatly reduce background and increase resolution, relative to conventional ChIP-seq.

The authors highlight the simplicity of their modifications and bioinformatic analysis relative to other recent modifications to ChIP-seq such as ChIP-exo and ChIP-nexus, making this of interest to the field. The authors aim to show the superiority of X-ChIP-seq through limited comparisons to other datasets (such as conventional ChIP, ChIP-exo, 3'NT, and DNaseI footprinting).

The major areas of concern are:

1) The claim that X-ChIP-seq provides “near base-pair resolution” is not convincingly supported and several of the comparisons with other datasets are incomplete (as detailed below), making it difficult to directly compare the X-ChIP-seq data with other techniques.

2) There is a severe lack of information provided on methods used for the processing and analysis of sequencing data. This makes it difficult to verify the validity of methods used and does not support the claim of “simple bioinformatic processing”.

Main text, first paragraph: Clarify the reasoning/evidence that this is “near base-pair resolution”? This is not clearly established in Skene et al. 2014 and is not readily apparent from Figure 1; Although the positioning in Figure 1B looks different from conventional ChIPseq, the width (resolution) of the peak looks very similar; Are the authors arguing that it is higher resolution because it is a closer match to the 3'NT data which is nucleotide resolution? (This should actually be called accuracy).

Related to Figure 1: Would it not be better to demonstrate the resolution if this technique at a single locus (rather than averaging all TSS) and/or with a DNA-binding protein that has a more defined position on DNA? See Figure3 CTCF.

What do ChIP-exo and ChIP-nexus look like for PolII?

Figure 1–figure supplement 1: It would be very helpful to show end positions for 1st reads of X-ChIP input for comparison. This would allow for clear demonstration of any reduction in of bias. Also, it is unclear if this analysis uses reads aligned to both strands; this could change interpretation of lower half of figure; left shift of + strand reads would correspond to a right shift of - strand reads. How would this affect the plots?

Main text, third paragraph: How would adding this step alter conventional ChIPseq? I.e. what fraction of reads from conventional ChIPseq are within these size ranges and what happens to resolution/accuracy when only these are analyzed? Again there is insufficient comparison between new and old technique. This is important for demonstrating an improvement and/or less bias.

Main text, fourth paragraph: clarify “unbiased approach” (at least in Methods) How many sites were used? What was their average size etc. (some of this information is in Figure 3 legend). Give source for DNase data.

Main text, fourth paragraph: clarify or show data to support the claim that “shorter fragments gave the highest resolution and smallest range in peak widths”; Again this would be better supported with a comparison of both X-ChIP-seq and conventional ChIP-seq (at least by bioinformatically selecting shorter insert sizes).

Main text, fifth paragraph: Clarify “achieve single nucleotide resolution” and comment on offset between the two techniques.

Main text, last paragraph: It would be helpful to see more analysis of low background as in many cases (i.e. transcription-related proteins that do not directly bind DNA) this could be as important as the claimed increase in resolution.

# Reply: Author response

**DOI:** 10.7554/eLife.09225.011

We believe that our initial submission did not clearly state the limitations of ChIP-seq and how the bias and length of fragments obtained by sonication prevent detailed analyses to provide high-resolution data. We have edited the manuscript to make it clear how the approaches used here to generate near single base-pair resolution are not applicable to conventional ChIp-seq.

*The major areas of concern are*:

*1) The claim that X-ChIP-seq provides “near base-pair resolution” is not convincingly supported and several of the comparisons with other datasets are incomplete (as detailed below), making it difficult to directly compare the X-ChIP-seq data with other techniques*.

The most direct evidence for near base-pair resolution is that we identify DNA fragment ends separated by 19 bp that precisely flank the 19 bp CTCF motif (Figure 4D). We now provide further comparisons to other datasets as described below.

*2) There is a severe lack of information provided on methods used for the processing and analysis of sequencing data. This makes it difficult to verify the validity of methods used and does not support the claim of “simple bioinformatic processing”*.

We thank the editors for pointing out these oversights. Further details of the bioinformatic processing, including calculation of half-height widths and generation of V-plots, are now included in the figure legends and the Materials and Methods section, as described below. In addition, we now refer to the detailed step-by-step library preparation protocol that we had previously published in Current Protocols in Molecular Biology (PMID: 25827087).

*Main text, first paragraph: Clarify the reasoning/evidence that this is “near base-pair resolution”? This is not clearly established in* *Skene et al. 2014* *and is not readily apparent from* *Figure 1**; Although the positioning in* *Figure 1B* *looks different from conventional ChIPseq, the width (resolution) of the peak looks very similar; Are the authors arguing that it is higher resolution because it is a closer match to the 3'NT data which is nucleotide resolution? (This should actually be called accuracy)*.

As pointed out above, the evidence for near base-pair resolution derives primarily from the fact that the 19 bp CTCF motif corresponded to fragment end maxima that were 19 bp apart, but also from the close correspondence of the PolII envelope obtained using our method to that obtained using 3’NT, which maps the single 3’ base in the active site of PolII. However, we have softened the statement by altering the text to:

“By using micrococcal nuclease (MNase) to digest unprotected DNA, we were able to achieve high resolution in a ChIP experiment, mapping the precise location of PolII and chromatin remodelers on the DNA (Figure 1A).”

In addition to the higher resolution, we also achieve higher accuracy, by avoiding sonication bias, as indicated by the close match to 3’NT. The text has been altered to include this discussion:

“In comparison to conventional ChIP-seq, using high-resolution X-ChIP-seq achieves both higher resolution, as indicated by the width of the ChIP peak and higher accuracy by avoiding sonication bias, as shown by high similarity to 3’NT.”

An intrinsic limitation of the 3’NT technique is that RNA has to be transcribed at least 25 nucleotides in length to be mapped and therefore 3’NT cannot determine PollII distribution upstream of +25bp relative to the TSS. The figure legend now points out this fact.

*Related to* *Figure 1**: Would it not be better to demonstrate the resolution if this technique at a single locus (rather than averaging all TSS) and/or with a DNA-binding protein that has a more defined position on DNA? See* *Figure3* *CTCF*.

We agree, although as PolII is a processive enzyme, which has a broad distribution along the length of genes, it is not ideal for this type of analysis. Rather, we analyzed CTCF at individual loci, because it binds to discrete sites.

*What do ChIP-exo and ChIP-nexus look like for PolII*?

There are no available ChIP-exo or ChIP-nexus datasets for PolII in *Drosophila* S2 cells. Despite ChIP-exo being first published in 2011, very few other labs have adopted the technique. Indeed the complexities of getting ChIP-exo to work seemed to be the motivation for developing a derivative of ChIP-exo: ChIP-nexus (“However, we found significant technical hurdles in applying ChIP-exo.” PMID: 25751057). This is why we used the example of CTCF to directly compare conventional ChIP-seq, high-resolution X-ChIP-seq and ChIP-exo.

*Figure 1–figure supplement 1**: It would be very helpful to show end positions for 1st reads of X-ChIP input for comparison. This would allow for clear demonstration of any reduction in of bias. Also, it is unclear if this analysis uses reads aligned to both strands; this could change interpretation of lower half of figure; left shift of + strand reads would correspond to a right shift of - strand reads. How would this affect the plots*?

Thank you for bringing up this point. We have now clarified Figure 1–figure supplement 1 to show the left ends (5’ position on forward strand reads) and right ends (3’ position on reverse strand reads) of fragments from single end sequencing data of sonicated input chromatin, which has been aligned to the transcriptional start site (reads corresponding to genes on the reverse strand were flipped).

A similar analysis of input chromatin from high-resolution X-ChIP-seq will not be comparable, as here the chromatin has been predominantly fragmented by MNase and therefore will provide footprints corresponding to nucleosomes and other DNA bound factors (PMID: 22025700). Indeed in a previous publication, we used mapping of the input chromatin to determine nucleosome positions (PMID: 24737864). The main text has been altered to include this:

“Analysis of a published sonicated input DNA sample indicates a strong sonication bias at the promoter region (Figure 1–figure supplement 1). In contrast, by predominantly fragmenting the chromatin with MNase it is possible to generate footprints corresponding to nucleosomes and other DNA-bound factors.”

*Main text, third paragraph: How would adding this step alter conventional ChIPseq? I.e. what fraction of reads from conventional ChIPseq are within these size ranges and what happens to resolution/accuracy when only these are analyzed? Again there is insufficient comparison between new and old technique. This is important for demonstrating an improvement and/or less bias*.

The application of this enrichment for short size classes to conventional ChIP-seq is not appropriate for two reasons: 1) The bias in sonication means that recovered DNA fragments in conventional ChIP-seq do not relate to the footprint of bound factors. Therefore, the length of the DNA fragments provides no information as to the location of the minimally protected footprint and as such, single end sequencing is appropriate for conventional ChIP-seq. 2) Typical sonication yields fragments between 200-500 bp, as indicated for the conventional ChIP-seq ENCODE CTCF dataset analyzed here (GSM749690) and even extensive sonication only further fragments chromatin down to an average of 200 bp (PMID: 18765474). Therefore, it is not possible to get high-resolution data from conventional ChIP-seq. The main text has been altered to include this discussion.

*Main text, fourth paragraph: clarify “unbiased approach” (at least in Methods) How many sites were used? What was their average size etc. (some of this information is in* *Figure 3* *legend). Give source for DNase data*.

Figure legend 3 has been altered to include these details:

“Sites were determined by identifying the DNase1 sites common to K562 and HeLa cells, as defined by the ENCODE project, that contained the 19 bp CTCF consensus binding motif (MA0139.1) by using FIMO analysis with a false discovery rate of 0.01. This identified 9403 such 19 bp CTCF motifs within DNase1 sites that were at least 500 bp apart.”

*Main text, fourth paragraph: clarify or show data to support the claim that “shorter fragments gave the highest resolution and smallest range in peak widths”; Again this would be better supported with a comparison of both X-ChIP-seq and conventional ChIP-seq (at least by bioinformatically selecting shorter insert sizes)*.

Figure 3B shows that smaller fragments provide the highest resolution as measured by the half-height width of the peak at each CTCF site. Here we show a comparison of the half-height widths for high-resolution X-ChIP-seq and conventional ChIP-seq. As discussed above, it is not possible to further separate conventional ChIP-seq by fragment sizes, as single end sequencing was used by the ENCODE project, which is appropriate as sonication yields 200-500 bp fragments, and because of the bias introduced by sonication the length of a fragment does not reflect its DNA footprint of protein binding.

To further illustrate this point, we have now included a V-plot analysis (PMID: 22025700) as a supplement, indicating that the shorter DNA fragments are more tightly grouped and closely centered over the CTCF motif. We have also included a discussion as to why this V-plot analysis is not possible for conventional ChIP-seq in the figure legend.

*Main text, fifth paragraph: Clarify “achieve single nucleotide resolution” and comment on offset between the two techniques*.

This statement was based upon the fact that by mapping the ends of the fragments captured by high-resolution X-ChIP-seq, we find that MNase chews back to give ends separated by 19 bp either side of the 19bp CTCF consensus motif (Figure 3D). The text has been altered to make this clearer. In addition, the x-axis of Figures 3D and Figure 3–figure supplement 2 have been altered to more clearly indicate that we are showing ChIP signal plotted at single nucleotide resolution.

The offset observed for DNaseI likely reflects the intrinsic differences in steric hindrance between DNase1/CTCF and MNase/CTCF to chromatin digestion. This is commented upon in the figure legend.

*Main text, last paragraph: It would be helpful to see more analysis of low background as in many cases (i.e. transcription-related proteins that do not directly bind DNA) this could be as important as the claimed increase in resolution*.

Here, we used the term background to reflect the large sprawling ChIP signal, which is observed in conventional ChIP-seq flanks the minimal DNA binding motif. This statement has been amended.
